# Activation of IP_3_R in atrial cardiomyocytes leads to generation of cytosolic cAMP

**DOI:** 10.1152/ajpheart.00152.2024

**Published:** 2024-08-02

**Authors:** Emily C. Akerman, Matthew J. Read, Samuel J. Bose, Andreas Koschinski, Rebecca A. Capel, Ying-Chi Chao, Milda Folkmanaite, Thamali Ayagama, Steven D. Broadbent, Rufaida Ahamed, Jillian N. Simon, Derek A. Terrar, Manuela Zaccolo, Rebecca A. B. Burton

**Affiliations:** ^1^Department of Pharmacology, University of Oxford, Oxford, United Kingdom; ^2^Department of Physiology, Anatomy and Genetics, University of Oxford, Oxford, United Kingdom; ^3^Axol Bioscience Limited, Cambridge, United Kingdom; ^4^Division of Cardiovascular Medicine, Radcliffe Department of Medicine, University of Oxford, Oxford, United Kingdom; ^5^Department of Pharmacology and Therapeutics, Institute of Systems, Molecular and Integrative Biology, University of Liverpool, Liverpool, United Kingdom

**Keywords:** adenylyl cyclase, Ca^2+^ signaling, cardiac atria, cyclic AMP, inositol trisphosphate

## Abstract

Atrial fibrillation (AF) is the most common sustained cardiac arrhythmia. Excessive stimulation of the inositol (1,4,5)-trisphosphate (IP_3_) signaling pathway has been linked to AF through abnormal calcium handling. However, little is known about the mechanisms involved in this process. We expressed the fluorescence resonance energy transfer (FRET)-based cytosolic cyclic adenosine monophosphate (cAMP) sensor EPAC-S^H187^ in neonatal rat atrial myocytes (NRAMs) and neonatal rat ventricular myocytes (NRVMs). In NRAMs, the addition of the α_1_-agonist, phenylephrine (PE, 3 µM), resulted in a FRET change of 21.20 ± 7.43%, and the addition of membrane-permeant IP_3_ derivative 2,3,6-tri-*O*-*butyryl*-myo-IP_3_(1,4,5)-hexakis(acetoxymethyl)ester (IP_3_-AM, 20 μM) resulted in a peak of 20.31 ± 6.74%. These FRET changes imply an increase in cAMP. Prior application of IP_3_ receptor (IP_3_R) inhibitors 2-aminoethyl diphenylborinate (2-APB, 2.5 μM) or Xestospongin-C (0.3 μM) significantly inhibited the change in FRET in NRAMs in response to PE. Xestospongin-C (0.3 μM) significantly inhibited the change in FRET in NRAMs in response to IP_3_-AM. The FRET change in response to PE in NRVMs was not inhibited by 2-APB or Xestospongin-C. Finally, the localization of cAMP signals was tested by expressing the FRET-based cAMP sensor, AKAP79-CUTie, which targets the intracellular surface of the plasmalemma. We found in NRAMs that PE led to FRET change corresponding to an increase in cAMP that was inhibited by 2-APB and Xestospongin-C. These data support further investigation of the proarrhythmic nature and components of IP_3_-induced cAMP signaling to identify potential pharmacological targets.

**NEW & NOTEWORTHY** This study shows that indirect activation of the IP_3_ pathway in atrial myocytes using phenylephrine and direct activation using IP_3_-AM leads to an increase in cAMP and is in part localized to the cell membrane. These changes can be pharmacologically inhibited using IP_3_R inhibitors. However, the cAMP rise in ventricular myocytes is independent of IP_3_R calcium release. Our data support further investigation into the proarrhythmic nature of IP_3_-induced cAMP signaling.

## INTRODUCTION

The contraction of cardiac muscle involves the coordination of multiple, precisely regulated signaling pathways, summarized by the process of excitation-contraction coupling (ECC) (1). ECC is initiated by depolarization of the sarcolemma in response to an action potential, resulting in calcium (Ca^2+^) influx across the sarcolemma, primarily through L-type Ca^2+^ channels (LTCCs). In ventricular myocytes, these are located within *t* tubules and positioned in proximity to the junctional space formed between the sarcolemma and the sarcoplasmic reticulum (1, 2), whereas atrial myocytes lack an extensive network of *t* tubules and LTCCs are primarily located in the surface membrane (3). In ventricular myocytes, ryanodine receptors (RyRs) in the terminal cisternae of the sarcoplasmic reticulum (SR) subsequently open in response to the rise in junctional Ca^2+^ accompanying opening of LTCCs, leading to further Ca^2+^ release from the SR. In atrial myocytes, activation of RyRs following Ca^2+^ entry via LTCCs occurs by a more complex mechanism described by Blatter (3) as a “fire-diffuse-fire” mechanism. Activation of Ca^2+^-induced Ca^2+^ release (CICR) in both ventricular and atrial myocytes provides an amplification of the original Ca^2+^ signal and a rise in overall cytosolic Ca^2+^, as represented by the rising phase of the cellular Ca^2+^ transient (4). These [Ca^2+^] transients (CaTs) can be recorded experimentally using intracellular Ca^2+^-sensitive dyes such as Fluo-5F. The falling phase of the CaT represents subsequent reuptake of Ca^2+^ into the SR via the sarco(endo)plasmic reticulum Ca^2+^-ATPase; release from the cell across the sarcolemma, primarily via the Na^+^/Ca^2+^ exchanger (NCX); or reuptake into mitochondria (1, 5, 6). ECC provides the link between electrical excitation of cardiac tissue, in the form of an action potential, and the rise in cytosolic Ca^2+^ leading to activation of the contractile actin and myosin filaments responsible for mechanical contraction of the heart tissue (7).

The regulation of ECC in ventricular myocytes is largely influenced by β-adrenergic and muscarinic receptor signaling (1). Activation of β-adrenergic receptors (β-ARs) leads to the downstream activation of adenylyl cyclases (ACs), primarily AC5 and AC6 (8, 9), and elevation of cyclic adenosine monophosphate (cAMP). This cAMP binds to the catalytic subunit of protein kinase A (PKA), leading to phosphorylation and activation of downstream effectors involved in controlling inotropy, including (but not limited to) LTCCs (10), phospholamban (11), RyRs (12), and cardiac troponin I (13, 14). In addition, Ca^2+^ handling in cardiac cells can be regulated by alternative second messengers, including inositol (1,4,5)-trisphosphate (IP_3_), Ca^2+^/calmodulin-dependent protein kinase II (CaMKII), cyclic adenosine diphosphate-ribose (cADPR), and nicotinic acid adenine dinucleotide phosphate (NAADP) (1, 7). Specificity of cAMP/PKA signaling is achieved through compartmentalization within specific subcellular nanodomains, primarily maintained via the action of phosphodiesterases, which hydrolyze cAMP to AMP, and the tethering of PKA to specific targets by A-kinase anchoring proteins (AKAPs) (15–17).

IP_3_ is a Ca^2+^-mobilizing second messenger that acts via IP_3_ receptors (IP_3_Rs) on the endoplasmic reticulum or SR to release Ca^2+^ in multiple cell types (18, 19). IP_3_ is known to play a role in Ca^2+^ handling in cardiac cells; however, in comparison with ECC and the role played by RyRs in CICR, the involvement of IP_3_ signaling in cardiac cellular Ca^2+^ handling is less well understood (20–25). Activation of G_q/11_-coupled receptors, including α-adrenergic receptors (α-ARs), angiotensin II receptor type 1, 5-hydroxytriptamine (5-HT) 2 receptor, and endothelin-1 receptors, leads to the cleavage of phosphatidylinositol-4,5 biphosphate (PIP_2_) to IP_3_, as well as diacylglycerol (26) and protein kinase C (PKC), via stimulation of phospholipase C (PLC) (18). In cardiomyocytes, binding of IP_3_ to IP_3_R on the SR leads to SR Ca^2+^ release (18, 19), enhancing cellular Ca^2+^ oscillations (27). However, when compared with the release of SR Ca^2+^ via RyRs, IP_3_R-mediated SR Ca^2+^ release is relatively slow, and insufficient to trigger the CICR cascade (28, 29). IP_3_R structure is partially homologous to that of RyR and comprises four identical subunits, each comprising ∼2,700 amino acids and containing an NH_2_-terminal IP_3_ binding sequence and other regulatory factors (23, 30, 31). In addition to IP_3_, IP_3_R may be regulated by cAMP/PKA and Ca^2+^ itself (32). In ventricular cells, IP_3_R is primarily located within the nuclear envelope (33), with some expression in *t* tubules (34), and is involved in the coupling of nuclear Ca^2+^ signaling to gene expression (35). In atrial cells, however, IP_3_R is expressed in the subsarcolemmal space, as well as the nuclear envelope, and may play a greater role in regulating Ca^2+^ handling and inotropy (21, 36). The spatial localization of IP_3_Rs within cells allows IP_3_R-mediated Ca^2+^ release to be specifically targeted to specific effectors within local signaling domains, including other IP_3_Rs, mitochondria, and lysosomes, as well as Ca^2+^-regulated channels and enzymes (32).

The precise role of IP_3_ and IP_3_R-mediated SR Ca^2+^ release in contributing to Ca^2+^ handling in cardiomyocytes remains unclear (7, 21, 29, 37). IP_3_ is positively inotropic in atrial (20), and ventricular tissue (38) and positively chronotropic in the sinoatrial node (SAN) (39, 40). IP_3_ and IP_3_R are involved in controlling pacemaker and spontaneous activity of cardiomyocytes in the embryonic heart (41–43), and in the diseased heart, the reemergence of gene expression profiles more closely replicating the embryonic condition results in IP_3_ having a more prominent role in these processes (23, 44, 45). Changes in the expression of IP_3_R have been linked with cardiac diseases, including atrial arrhythmias and heart failure (20, 46, 47). Expression of IP_3_R type 2 (IP_3_R2), the most abundant IP_3_R isoform in cardiac tissue (20, 39), is at least six times greater in the atria compared with the ventricle (20), and IP_3_R expression is upregulated in both human patients with chronic atrial fibrillation (AF) (46) and canine AF models (47). Elevated Ca^2+^ and Ca^2+^ oscillations resulting from IP_3_R Ca^2+^ release could contribute to arrhythmogenesis (29), for example, through the stimulation of transient inward current (48), leading to delayed after depolarizations (49, 50); by delaying repolarization and increasing the likelihood of reentry (51); or by slowing conduction (29, 52). IP_3_ is upregulated in patients with end-stage heart failure, coinciding with a downregulation of RyRs (37), and IP_3_R expression increases with age (21, 37, 53), an observation that parallels age-related AF disease progression from paroxysmal to persistent forms (21, 54, 55).

The inotropic and chronotropic effects of IP_3_ in atrial tissue are dependent on AC and PKA activity, as shown by the inhibition of IP_3_-mediated changes in atrial contractility and spontaneous beating rate by the nonspecific AC inhibitor MDL-12330A and the PKA inhibitor H89 (25). These effects are independent of β-AR activation and remain in the presence of the β-AR inhibitor metoprolol (25). It has been suggested that the effects of IP_3_R-mediated Ca^2+^ release on cardiomyocyte contractility result from increased [Ca^2+^] within the vicinity of RyRs, thereby enhancing the response of RyRs to Ca^2+^ influx via LTCCs (23, 56). However, the observation that changes in CaT amplitude in response to IP_3_-producing agonists are inhibited by MDL-12330A and H89 implicates a role for ACs downstream of IP_3_R activation and independent of direct RyR sensitization (25).

A possible explanation for the involvement of AC activity downstream of IP_3_R Ca^2+^ release is the direct stimulation of Ca^2+^-activated AC isoforms, AC1 or AC8, which are both expressed in proximity to IP_3_R in atrial and SAN cells (25, 57–59) and are thought to influence SAN pacemaker activity through an influence on the pacemaker current (*I*_f_) (60). Increases in SAN beating rate in response to activation of α-AR by phenylephrine (PE) are dependent on AC1 activity, as shown using the AC1-selective inhibitor ST034307 (59); however, it remains unclear whether Ca^2+^-activated ACs are, directly or indirectly, involved in the downstream effects of IP_3_ signaling or whether activation of IP_3_R leads to a Ca^2+^-dependent activation of AC and localized changes in cAMP within cardiomyocytes. Fluorescence resonance energy transfer (FRET)-based sensors enable real-time measurement of intracellular cAMP signaling and can be used to demonstrate compartmentalization of cAMP/PKA signaling (16, 17, 61). Cytosolic sensors, such as EPAC-S^H187^, enable global changes in cytosolic cAMP to be measured from intact cells in real time using fluorescent imaging (62), whereas fusion of PKA/cAMP FRET-based sensors to specific targeting domains such as AKAPs can be used to measure localized cAMP changes to determine compartmentalization of cAMP/PKA signaling events within subcellular nanodomains (16, 17, 61, 63). Localization of cAMP/PKA signaling, resulting from the tethering of PKA by anchoring proteins such as AKAPs (64, 65) and β-arrestins (66, 67), as well as compartmentalization within caveolae (68, 69), accounts for the specificity of responses to cAMP. The purpose of the present study was to use a combination of FRET-based reporters, including the cytosolic sensor EPAC-S^H187^, and plasma membrane-localized AKAP79-CUTie, in combination with specific IP_3_R inhibitors, to determine whether activation of the IP_3_ signaling pathway in cardiomyocytes is linked to localized cAMP signals.

## MATERIALS AND METHODS

All experiments were performed in accordance with the United Kingdom Home Office’s *Guide on the Operation of Animal (Scientific Procedures) Act 1986*.

### Cell Isolation and Infection

Neonatal rat atrial myocytes (NRAMs) were isolated using a modified protocol based on previous methods used to isolate neonatal rat ventricular myocytes (NRVMs) (70, 71). Hearts were isolated from 3-day-old Sprague-Dawley rat pups, culled by cervical dislocation. Following dissection, atria were separated from the ventricles, transferred to 2 mL of Dulbecco’s modified Eagle’s medium (DMEM), and cut into 1–2 mm^3^ pieces. Atrial myocytes were enzymatically isolated by a series of enzymatic digestions, in trypsin (1 mg/mL, Merck, UK, rocked at 4°C for 2 h) followed by collagenase (type IV, 1 mg/mL, Merck, UK), as described in the work by Burton et al. (70). For the collagenase digestions, the trypsin was replaced by 4 mL of collagenase solution and the tissue was gently triturated using a plastic pipette for 1 min. The tissue plus collagenase solution was then stirred gently in a bath at 37°C for 2 min, and the first supernatant was discarded. A further 4 mL of collagenase solution was added to the tissue pellet and triturated for 1 min using a wide-bore pipette. This solution was then stirred gently in a bath at 37°C for 2 min, and the supernatant (4 mL) was added to 3 mL of Hanks’ buffered salt solution and stored in a 15-mL centrifuge tube. This process was repeated a further three times to produce a total of 4 × 7-mL cell suspensions. Tubes were centrifuged at 2,000 rpm for 8 min. The supernatant was then removed, and the cells were resuspended in 2 mL of cardiomyocyte plating media (CPM), consisting of 85% DMEM, 17% M199, 10% horse serum, 5% FBS, and 1% penicillin-streptomycin, before being centrifuged at 100 *g* for 10 min. All samples were then strained using a cell strainer and pooled into a single 50-mL centrifuge tube. Isolated cells were preplated and incubated at 37°C (95% O_2_–5% CO_2_) for 1 h to allow for the separation of fibroblasts. The supernatant containing suspended atrial myocytes was then removed, and cell density was measured using a hemocytometer and trypan blue. Myocytes were seeded onto 24-mm glass coverslips coated with 450 µL of laminin (40 μg/mL, Merck, UK) in 35-mm six-well plates at a density of 15,000 cells/mL in CPM. NRVMs were isolated as previously described (61). Cells were infected with EPAC-S^H187^ or AKAP79-CUTie second-generation adenoviral vectors after 72 h and incubated at 37°C (72). Imaging was carried out 24–36 h after infection; the experimental timeline is described in Supplemental Fig. S1 (Supplemental material may be accessed at https://doi.org/10.6084/m9.figshare.26335852.v1).

### FRET Imaging

FRET imaging was performed in an organ bath at room temperature 24 h after infection using an inverted microscope (Olympus IX71) with a PlanApoN, 60, NA 1.42 oil immersion objective, 0.17/FN 26.5 (Olympus, UK) attached to a coolSNAP HQ^2^ camera and a DV2 optical beam splitter (MAG Biosystems, Photometrics, UK) for simultaneous recording of YFP and CFP emissions. Cells were maintained at room temperature in an extracellular solution (E1), consisting of (in mM) 140 NaCl, 3 KCl, 2 MgCl_2_, 2 CaCl_2_, 15 glucose, and 10 HEPES (pH 7.2 with NaOH). Pipetting time control experiments were conducted to ensure results were not confounded by the solvent or time. Pipetting time control consisted of the addition of either E1 on its own (for PE) or dimethylsulfoxide (DMSO) (solvent for 2-APB and Xestospongin-C) and 0.0001% pluronic F127 with 0.009% DMSO (solvent for IP_3_-AM) dissolved in E1. Images were acquired and processed using MetaFluor 7.1 (Meta Imaging Series, Molecular Devices). FRET ratio changes were measured as changes of the background-subtracted 480-nM/545-nM fluorescence (cyan/yellow) emission intensity on excitation at 430 nM and expressed as *R*/*R*_0_, where *R* is the ratio at time *t* and *R*_0_ is the average ratio of the first 240 s. For each plate of cells, multiple cells were analyzed individually within the field of view, and the mean FRET ratio (*n* = 1) was calculated as the average for all cells within the field of view.

### Multielectrode Array

For multielectrode array (MEA) experiments, human induced pluripotent stem cells-derived atrial cardiomyocytes (hiPSC-ACMs) (ax2518, Axol Bioscience, UK) were cultured at 5.0 × 10^5^ cells/well on a 768-channel 48-well Axion Cytoview MEA 48 plate (M768-tMEA-48B, Axion Biosystems, USA) coated with Axol’s Fibronectin Coating Solution (ax0049, Axol Bioscience, UK) at 37°C in a 5% CO_2_–95% air atmosphere. Cells were cultured to *day 19* after plating, and all experiments were performed on *day 19*. Spontaneous extracellular field potentials were acquired at 37°C using a high-throughput Maestro Pro MEA system and AxIS software (v.2.4, Axion Biosystems). Extracellular potentials were simultaneously recorded for 16 channels per well across 48-well plates at a sampling rate of a 12.5-kHz channel. Synchronized, spontaneous beats were first observed on most electrodes across all wells from 72 h post-thaw. For experiments, control activity, both field action potential (FAP) and contractility, was recorded for 5 min before the addition of DMSO, H89 (1 μM), or MDL-12330A (3 μM). Following the addition, FAP recordings were made for 15 min, followed by contractility recording for 5 min. PE was then added at increasing concentrations from 1 to 30 μM, and FAP followed by contractility recordings were made following each subsequent addition of PE for periods of 5 min (total recording time at each concentration of PE = 10 min). Recordings were made from an additional 12 wells throughout the experimental period as an environmental control.

### Data Analysis and Statistics

Data are presented as means ± SD, and statistical analysis was performed using GraphPad Prism (v.10.1.1). Data from multiple individual cells within the field of view from a single plate were averaged to give *n* = 1. Data are represented as *n* = number of biological replicates and *N* = number of litters from different rat mothers used for neonatal isolations (10–14 pups per litter), representing the number of independent isolations conducted from litters. Between *n* = 1–3 biological replicates were conducted for *N* = 1 neonatal isolation. For FRET experiments, data were assumed to be non-Gaussian. To compare two datasets, a Mann–Whitney test or Student’s *t* test was used. Otherwise, Kruskal–Wallis followed by Dunn’s multiple-comparisons or Friedman tests were used to compare data for unpaired (Kruskal–Wallis) or paired (Friedman’s) data, as appropriate. Statistical significance, when achieved, is indicated as *P* < 0.05, *P* < 0.01, *P* < 0.001, and *P* < 0.0001.

## RESULTS

### Phenylephrine Causes a Rise in Cytosolic cAMP in Cultured NRAMs

**Figure 1.**
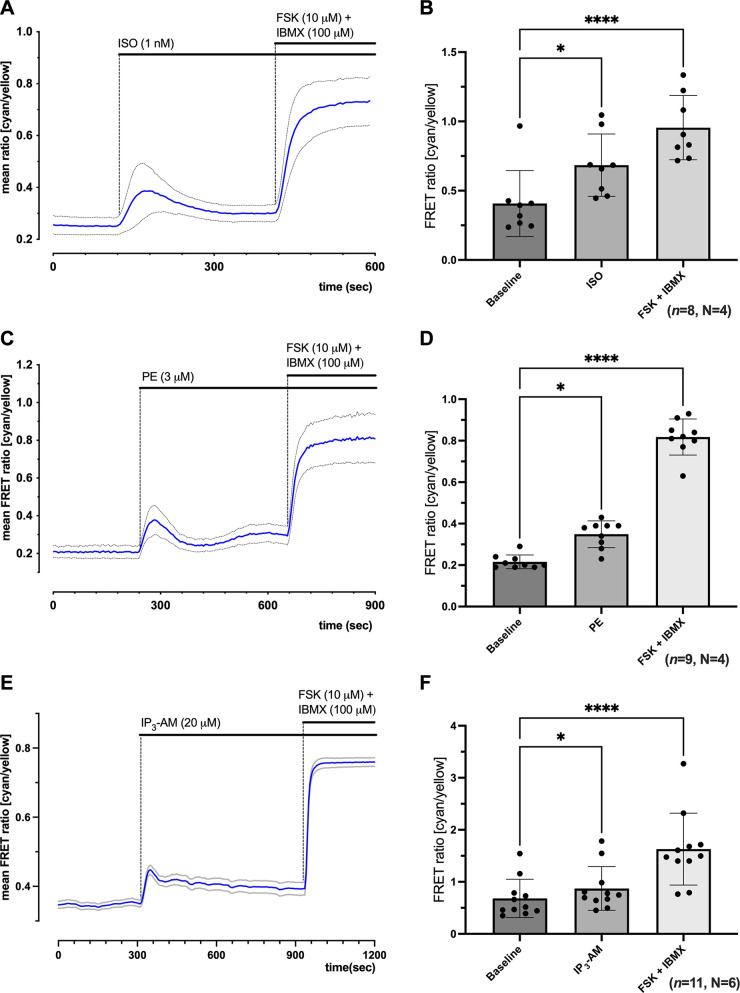
Activation of inositol trisphosphate (IP_3_) signaling pathway in neonatal rat atrial myocytes (NRAMs) using phenylephrine (PE) results in an increase in cytosolic cyclic adenosine monophosphate (cAMP), as shown using the EPAC-S^H187^ fluorescence resonance energy transfer (FRET) sensor. *A*: representative trace from single plate (7 cells) showing time course for change in the mean FRET ratio (cyan/yellow) on the addition of 1 nM isoprenaline (Iso) followed by 10 μM forskolin (FSK) + 100 μM 3-isobutyl-1-methylxanthine (IBMX). *B*: quantification of total data showing the mean FRET ratio at baseline, peak response to 1 nM Iso, and saturating response to 10 μM FSK + 100 μM IBMX (*n* = 8, *N* = 3). *C*: representative trace from single plate (7 cells) showing time course for change in the mean FRET ratio (cyan/yellow) on the addition of 3 μM PE followed by 10 μM FSK + 100 μM IBMX. *D*: quantification of total data showing the mean FRET ratio at baseline, peak response to 3 μM PE, and saturating response to 10 μM FSK + 100 μM IBMX (*n* = 9, *N* = 4). *E*: representative time course from single plate (*n* = 8 cells) showing time course for change in the mean FRET ratio (cyan/yellow) on the addition of 20 μM membrane-permeant *myo*-inositol-145-trisphosphate derivative (IP_3_-AM) followed by 10 μM FSK + 100 μM IBMX. *F*: quantification of total data showing the mean FRET ratio at baseline, peak response to 20 μM IP_3_-AM, and saturating response to 10 μM FSK + 100 μM IBMX (*n* = 11, *N* = 6). Representative traces and FRET ratios are represented as means ± SD. Black circles, individual data points; ns, not significant. *P < 0.05 and ****P < 0.0001, 1-way ANOVA followed by uncorrected Dunn’s test.

To test the response of NRAMs expressing the cytosolic cAMP FRET sensor EPAC-S^H187^ to β-AR stimulation, we used the β-AR agonist isoprenaline (Iso, 1 nM). Figure 1*A* shows a representative time course for FRET change, representing cytosolic cAMP levels, on application of 1 nM Iso to the extracellular bath solution followed by a sensor saturating concentration of forskolin (FSK, 10 μM) and 3-isobutyl-1-methylxanthine (IBMX, 100 μM). Individual traces for the CFP and YFP intensity curves corresponding to the experiment shown in Fig. 1*A* are presented in Supplemental Fig. S2*A.* Extracellular application of 1 nM Iso to NRAMs expressing EPAC-S^H187^ resulted in an increase in the mean FRET ratio (cyan/yellow) from 0.27 ± 0.01 to a peak of 0.56 ± 0.04, representing a FRET ratio change of 44% of the saturating FSK/IBMX response (Fig. 1*A*). Quantification of the FRET ratio change in response to Iso (1 nM) and the saturating stimulus compared with baseline is shown in Fig. 1*B*. In contrast, the addition of the α_1_-AR agonist PE (3 μM) resulted in a peak corresponding to 0.35 ± 0.02 of the saturating response, representing a FRET ratio change of 21% of the saturating response (Fig. 1*C*; Supplemental Fig. S2*B*). Quantification of the peak FRET ratio change in response to PE (3 µM, *n* = 9, *N* = 4) and the saturating stimulus compared with the baseline is shown in Fig. 1*D*. PE was tested at a range of concentrations from 1 to 10 μM, and the maximal change in the FRET ratio for these experiments is shown in Supplemental Fig. S2, *C–E*. Based on these results, a concentration of 3 μM PE was used for subsequent experiments. The peak FRET change response to PE (21.20 ± 7.43%, *P* < 0.0001) corresponded to a significant increase when compared with the pipetting time control, with E1 on its own (1.47 ± 0.67%, *n* = 6, *N* = 3) (see Supplemental Fig. S2*F*).

As activation of α-ARs using PE may result in the activation of alternative signaling pathways via activation of PKC and diacylglycerol (18, 26) and to rule out the possibility that the observed changes in cAMP resulted from G_s_-α-subunit-mediated adenylyl cyclase activation following β-AR activation by PE, we carried out additional experiments using the membrane-permeant IP_3_ derivative, 2,3,6-tri-*O*-*butyryl*-*myo*-IP_3_(1,4,5)-hexakis(acetoxymethyl)ester (IP_3_-AM). We found that the addition of IP_3_-AM (20 μM) to NRAMs expressing EPAC-S^H187^ produced a peak in the FRET ratio (Fig. 1*E*). Individual traces for the CFP and YFP intensity curves corresponding to the experiment shown in Fig. 1*E* are presented in Supplemental Fig. S2*G*. Comparison of the mean FRET ratio resulted in a significant peak of 0.87 ± 0.13 from baseline (0.68 ± 0.11) following the addition of IP_3_-AM (20 μM, *n* = 11, *N* = 6, *P* < 0.05) (Fig. 1*F*). The peak FRET changes in response to IP_3_-AM (21.31 ± 7.43 vs. 3.61 ± 2.04%, *P* < 0.01) were significant when compared with the pipetting time control, 0.0001% pluronic F127 with 0.009% DMSO (*n* = 6, *N* = 4) (Supplemental Fig. S2*H*).

### Pharmacological Inhibition of IP_3_R Prevents Rises in Cytosolic cAMP in Response to Phenylephrine and IP_3_-AM

**Figure 2.**
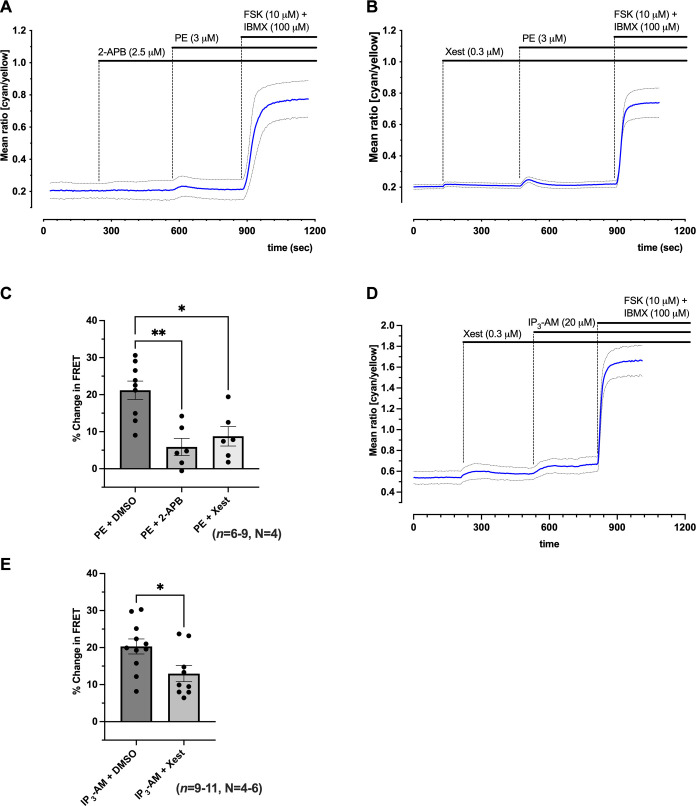
Inhibition of inositol trisphosphate receptor (IP_3_R) in neonatal rat atrial myocytes (NRAMs) prevents rise in cytosolic cyclic adenosine monophosphate (cAMP) in response to phenylephrine (PE). *A* and *B*: representative time course from single plate [6 cells (*A*), 4 cells (*B*)] showing time course for change in the mean fluorescence resonance energy transfer (FRET) ratio (cyan/yellow) on the addition of 3 μM PE followed by 10 μM forskolin (FSK) + 100 μM 3-isobutyl-1-methylxanthine (IBMX) after the initial addition of either 2.5 μM 2-aminoethoxydiphenyl borate (2-APB; *A*) or 0.3 μM Xestospongin-C (*B*). *C*: quantification of PE (3 μM; *n* = 9, *N* = 4) alone, in the presence of 2.5 μM 2-APB (*n* = 6, *N* = 4) or 0.3 μM Xestospongin-C (*n* = 6, *N* = 4), expressed as a fraction of the saturating response in the presence of 10 μM FSK + 100 μM IBMX. *D*: representative time course from single plate (3 cells) showing time course for change in the mean FRET ratio (cyan/yellow) on the addition of 20 μM membrane-permeant *myo*-inositol-145-trisphosphate derivative (IP_3_-AM) followed by 10 μM FSK + 100 μM IBMX after the initial addition of 0.3 μM Xestospongin-C. *E*: quantification of peak responses for control vehicle (*n* = 6, *N* = 3) or IP_3_-AM (20 μM; *n* = 11, *N* = 6) alone, or in the presence of 0.3 μM Xestospongin-C (*n* = 9, *N* = 4), expressed as a fraction of the saturating response in the presence of 10 μM FSK + 100 μM IBMX. Data are represented as means ± SD. Black circles, individual data points; ns, not significant. *P < 0.05 and **P < 0.01, Kruskal–Wallis followed by Dunn’s multiple-comparisons test (*C*) or unpaired Student’s *t* test (*D*).

To determine whether the changes in cytosolic cAMP recorded in response to IP_3_-AM and PE (Fig. 1) occur downstream of IP_3_R activation, we used the IP_3_R inhibitors 2-aminoethyl diphenylborinate (2-APB) and Xestospongin-C (Fig. 2). Figure 2, *A* and *B*, shows representative time courses for the change in FRET recorded from NRAMs expressing EPAC-S^H187^ in response to PE (3 μM) in the presence of either 2-APB (2.5 μM, Fig. 2*A*) or Xestospongin-C (0.3 μM, Fig. 2*B*). Individual traces for the CFP and YFP intensity curves corresponding to the experiment shown in Fig. 2, *A* and *B*, are presented in Supplemental Fig. S3, *A* and *B*. Quantification of the effect of 2-APB and Xestospongin-C on the response to PE compared with PE applied in the absence of IP_3_R inhibition is shown in Fig. 2*C*. Prior application of 2-APB (2.5 μM) inhibited the peak change in FRET following the addition of PE (3 μM) from 21.20 ± 7.43% (*n* = 9, *N* = 4) to 5.89 ± 5.66% (*n* = 6, *N* = 4, *P* < 0.01), whereas Xestospongin-C reduced the peak to 8.76 ± 6.43% (*n* = 6, *N* = 4, *P* < 0.05) (Fig. 2*C*).

Figure 2*D* shows the representative time course for the change in the FRET ratio recorded from NRAMs expressing EPAC-S^H187^ in response to IP_3_-AM (20 μM) in the presence of Xestospongin-C (0.3 μM). Individual traces for the CFP and YFP intensity curves corresponding to the experiment shown in Fig. 2*D* are presented in Supplemental Fig. S3*C*. Quantification of the effect of Xestospongin-C on the peak response to IP_3_-AM compared with IP_3_-AM applied in the absence of IP_3_R inhibition is shown in Fig. 2*E*. Prior application of Xestospongin-C (*n* = 9, *N* = 4) reduced the peak response to IP_3_-AM to 12.97 ± 6.48% (*n* = 9, *N* = 4, *P* < 0.05) compared with DMSO 21.31 ± 7.43% (Fig. 2*E*). Xestospongin-C (*n* = 21, *N* = 10) alone had no effect on the cytosolic FRET signal before the addition of IP_3_-AM or PE compared with control (*n* = 34, *N* = 15) (Supplemental Fig. S3*D*) (2.36 ± 5.74 vs. 2.41 ± 4.42%, *P* > 0.9999), and cAMP levels in response to either PE or IP_3_-AM were observed to be significantly reduced when IP_3_R Ca^2+^ release was blocked.

### Activation of cAMP Downstream of IP_3_R in Cultured Neonatal Ventricular Cardiomyocytes

**Figure 3.**
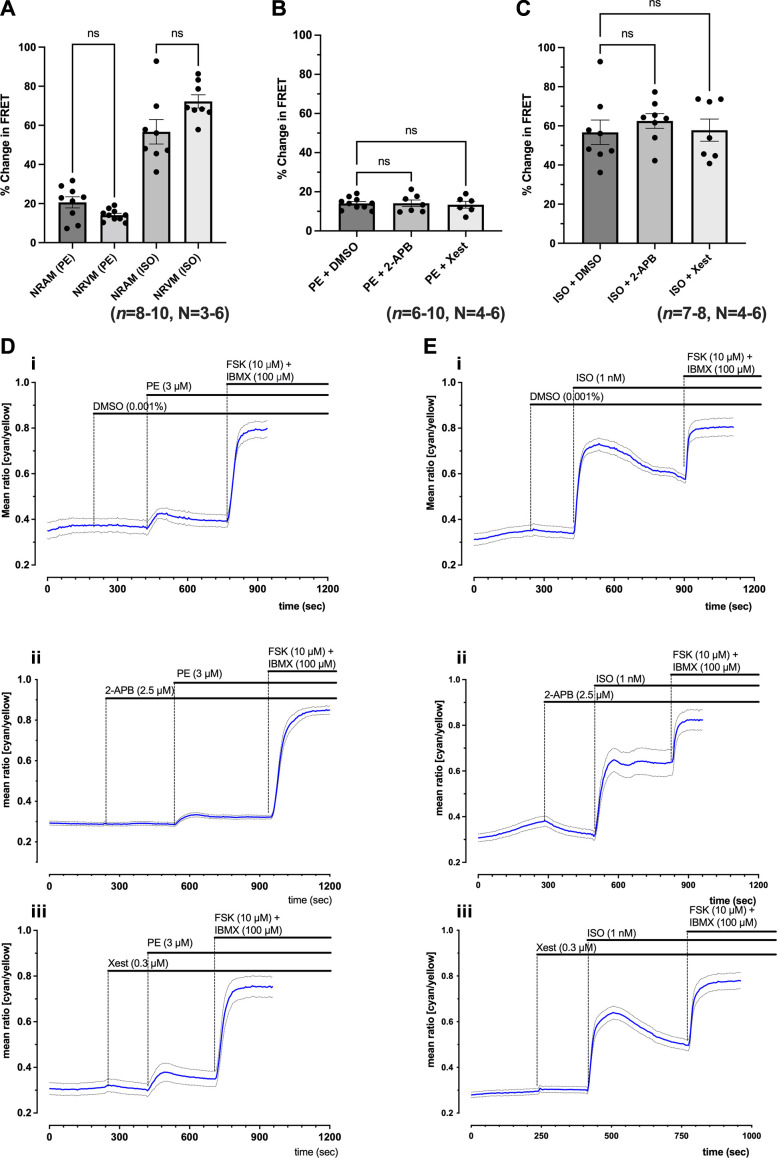
Phenylephrine (PE) and isoprenaline (Iso) stimulation on cytosolic cyclic adenosine monophosphate (cAMP) levels recorded in ventricular myocytes expressing EPAC-S^H187^ fluorescence resonance energy transfer (FRET) sensor show that response to PE is not affected by inositol trisphosphate receptor (IP_3_R) inhibition in ventricular cells. *A*: comparison of peak response to 3 μM PE or 1 nM Iso in neonatal rat atrial myocytes (NRAMs) and neonatal rat ventricular myocytes (NRVMs), shown as percent FRET change from baseline as normalized to saturating forskolin (FSK)/3-isobutyl-1-methylxanthine (IBMX) (*n* = 8–10, *N* = 3–6). *B*: quantification of the peak FRET change in response to 3 μM PE in NRVMs in the presence of 1 μL/mL DMSO, 2.5 μM 2-aminoethoxydiphenyl borate (2-APB) or 0.3 μM Xestospongin-C in NRVMs (*n* = 6–10, *N* = 4–6). *C*: quantification of the peak FRET change in response to 1 nM Iso in NRVMs in the presence of 1 μL/mL dimethylsulfoxide (DMSO), 2.5 μM 2-APB or 0.3 μM Xestospongin-C in NRVMs (*n* = 7–8, *N* = 4–6). *D*: representative traces from single plates showing time course for change in the mean FRET ratio (cyan/yellow) on the addition of 1 nM Iso followed by 10 μM FSK + 100 μM IBMX in control conditions (7 cells, *i*) or the presence of 2.5 μM 2-APB (8 cells, *ii*) or 0.3 μM Xestospongin-C (13 cells, *iii*) in NRVMs. *E*: representative traces from single plates showing time course for change in the mean FRET ratio (cyan/yellow) on the addition of 3 μM PE followed by 10 μM FSK + 100 μM IBMX in control conditions (6 cells, *i*) or the presence of 2.5 μM 2-APB (14 cells, *ii*) or 0.3 μM Xestospongin-C (8 cells, *iii*) in NRVMs. Data show means ± SD. Kruskal–Wallis followed by Dunn’s multiple-comparisons test. ns, not significant. *P* > 0.05.

As IP_3_R expression has been shown to be greater in atrial cells compared with ventricular cells (20), we were interested in determining whether cAMP changes in response to α-adrenergic stimulation would differ in NRVMs compared with NRAMs. As shown in Fig. 3*A*, a rise in cytosolic cAMP was observed in NRVMs expressing EPAC-S^H187^ in response to PE, with an amplitude of 14.07 ± 2.97% (*n* = 10, *N* = 6). The rise in cAMP observed in response to PE in NRVMs did not differ from the rise observed in response to PE in NRAMs (*P* > 0.05, Fig. 3*A*). In addition to PE, experiments using Iso (1 nM) were also performed. The rise in cAMP observed in response to 1 nM Iso was also not significantly altered in NRVMs (72.26 ± 9.59%, *n* = 8, *N* = 6) compared with NRAMs (53.97 ± 14.84%, *n* = 8, *N* = 3) (*P* > 0.05, Fig. 3*A*). In contrast to NRAMs (Fig. 2), rises in cAMP in response to PE and Iso in NRVMs were not found to undergo inhibition by either 2-APB or Xestospongin-C (Fig. 3, *B* and *C*). The representative traces in Fig. 3, *D*, *i–iii*, and *E*, *i–iii*, show the effects of PE and Iso on NRVMs in the presence of 2.5 μM 2-APB and 0.3 μM Xestospongin-C. Individual traces for the CFP and YFP intensity curves corresponding to the experiments shown in Fig. 3 are presented in Supplemental Fig. S4.

### Rises in cAMP in Response to Phenylephrine Are Localized at the Plasma Membrane in Neonatal Rat Atrial Cardiomyocytes

**Figure 4.**
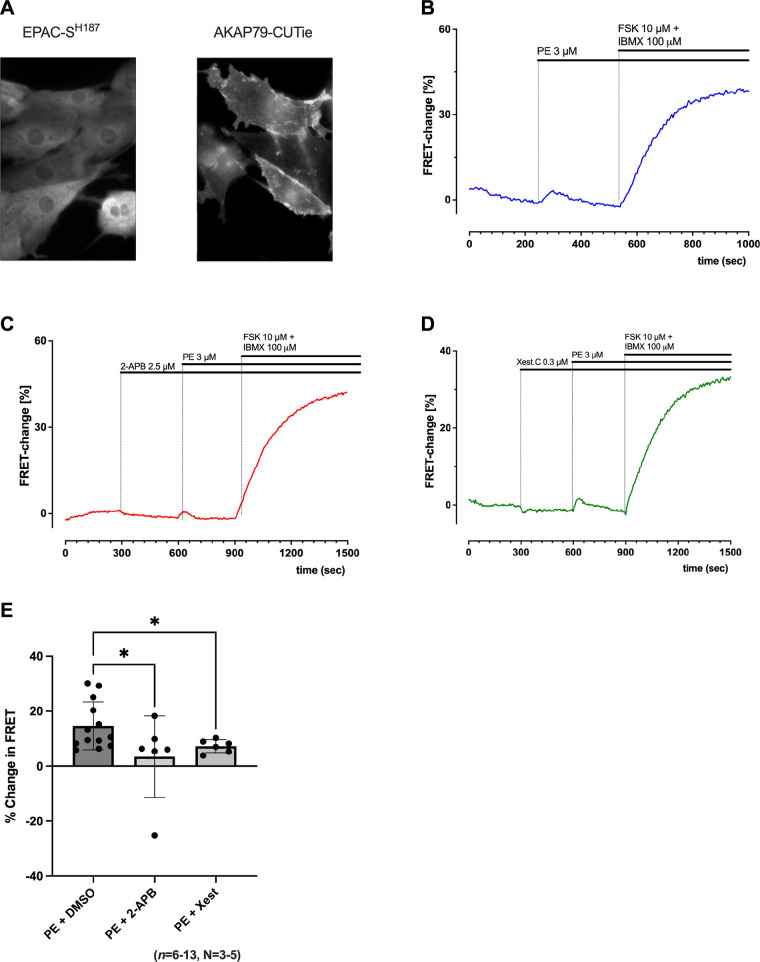
Increases in cyclic adenosine monophosphate (cAMP) in response to inositol trisphosphate receptor (IP_3_R) activation are localized at the plasma membrane, as shown using the AKAP79-CUTie fluorescence resonance energy transfer (FRET) sensor. *A*: images showing representative staining in neonatal rat atrial myocytes (NRAMs) for EPAC-SH^187^ and AKAP79-CUTie FRET sensors. *B*: representative trace showing time course for change in the FRET ratio (yellow/cyan) recorded from cells expressing AKAP79-CUTie on the addition of 3 μM phenylephrine (PE) followed by 10 μM forskolin (FSK) + 100 μM 3-isobutyl-1-methylxanthine (IBMX) (representative trace from single cell). *C* and *D*: representative trace showing time course for change in the FRET ratio (yellow/cyan) recorded from cells expressing AKAP79-CUTie on the addition of 2.5 μM 2-aminoethoxydiphenyl borate (2-APB; *C*) or 0.3 μM Xestospongin-C (*D*), followed by 3 μM PE and 10 μM FSK + 100 μM IBMX (representative trace from single cell). *E*: quantification of peak response to PE (3 μM; *n* = 13, *N* = 5) alone, or in the presence of 2.5 μM 2-APB (*n* = 5, *N* = 3) or 0.3 μM Xestospongin-C (*n* = 6, *N* = 3), expressed as a fraction of the saturating response in the presence of 10 μM FSK + 100 μM IBMX. Data are represented as means ± SD. Black circles, individual data points. *P < 0.05 and ***P < 0.001, Kruskal–Wallis followed by Dunn’s multiple-comparisons test (*E*).

One hypothesis previously presented for the cAMP-dependent response to IP_3_R activation in atrial cells is the involvement of membrane-bound Ca^2+^-sensitive AC1 or AC8 (25). We were, therefore, interested to test whether local changes were observed in cAMP activity at the plasma membrane in response to α-adrenergic stimulation by using cells expressing the plasma membrane-bound FRET sensor AKAP79-CUTie. AKAP79-CUTie is targeted to the AKAP79/β-AR/AC/LTCC complex located at the internal surface of the plasmalemma facing the intracellular space (16), and AKAPs have been linked to forming complexes with ACs (73). Figure 4*A* shows representative images comparing the distribution of the cAMP signal of the cytosolic EPAC-S^H187^ FRET sensor and AKAP79-CUTie. The cAMP signal detected by AKAP79-CUTie was reduced compared with EPAC-S^H187^, which would also be expected because of the lower affinity of AKAP79-CUTie for cAMP compared with EPAC-S^H187^ (72), and showed localization at the periphery of the cell, as would be expected to coincide with AKAP79 expression and in agreement with data previously reported (16).

Figure 4*B* shows a representative trace indicating the change in the FRET signal from AKAP79-CUTie expressed in NRAMs on the addition of PE (3 μM) followed by FSK (10 μM) and IBMX (100 μM). Figure 4, *C* and *D*, shows representative traces for the change in FRET signal observed in NRAMs expressing AKAP79-CUTie in response to 3 μM PE in the presence of either 2-APB (2.5 μM, Fig. 4*C*) or Xestospongin-C (0.3 μM, Fig. 4*D*) followed by FSK (10 μM) and IBMX (100 μM). As shown in Fig. 4*E*, cells expressing AKAP79-CUTie showed a 14.64 ± 8.71% increase in global FRET signal on the addition of PE (*P* < 0.05, *n* = 13, *N* = 5), indicating a rise in cAMP at the location of AKAP79/β-AR/AC/LTCC complex. 2-APB was found to cause a significant reduction in the change in FRET signal corresponding to 3.45 ± 14.7% (*n* = 6, *N* = 3) in response to PE compared with PE alone (Fig. 4*E*, P < 0.001). Although Xestospongin-C resulted in a smaller reduction in the change of FRET corresponding to 7.24 ± 2.38% (*n* = 6, *N* = 3) in response to PE, a significant reduction was still observed (*P* < 0.05).

#### Pharmacological inhibition of AC and PKA in hiPSC-ACMs inhibits the response to phenylephrine.

**Figure 5.**
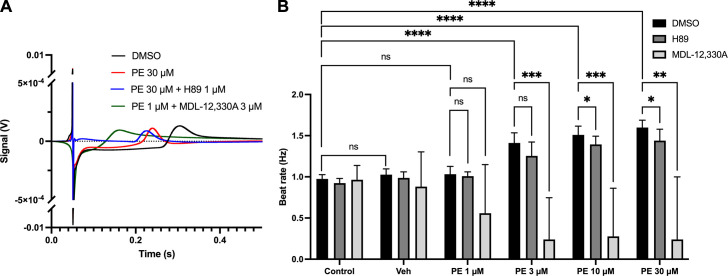
Inositol trisphosphate (IP_3_) pathway is active in human atrial induced pluripotent stem cells (iPSCs) as shown using multielectrode array. *A*: representative averaged multielectrode array (MEA) field action potential waveforms from control (dimethylsulfoxide, DMSO; *n* = 7), phenylephrine (PE; 30 μM; *n* = 11), and PE (1 or 30 μM) in the presence of either H89 (1 μM; *n* = 7) or MDL-12330A (3 μM; *n* = 1–10). *B*: effect of H89 (1 μM) and MDL-12330A (3 μM) on the beat rate of human iPSCs-derived atrial cardiomyocytes (hiPSC-ACMs) in response to PE at concentrations of 1–30 μM (DMSO, *n* = 7; H89, *n* = 7; MDL-12330A, *n* = 10). Data are represented as means ± SD. *P < 0.05, **P < 0.01, and ***P < 0.001, 2-way repeated-measures ANOVA followed by Sidak’s multiple comparisons. IP_3_, inositol (1,4,5)-trisphosphate.

Previous studies investigating the role of cAMP signaling in response to IP_3_R activation (25, 59) have been limited to the use of animal experiments, including intact mouse atria and isolated guinea pig atrial myocytes. To demonstrate the potential translational relevance of this pathway and the potential involvement of AC and PKA in the response to PE in human atrial cells, we performed multielectrode array (MEA) experiments using human-derived hiPSC-ACMs. Figure 5*A* shows representative averaged MEA field action potential waveforms from control (DMSO), PE (30 μM), and PE (1 or 30 μM) in the presence of either H89 (1 μM) or MDL-12330A (3 μM). Traces have been zoomed in to highlight differences in T wave and QT interval and demonstrate consistent shortening of the Q-T interval in the presence of PE. As shown in Fig. 5*B*, the addition of PE at concentrations of 3, 10, and 30 μM caused a successive increase in beat rate (*n* = 7), which was inhibited by H89 at high concentrations of PE (10 and 30 μM PE; *n* = 7; *P* < 0.05). The addition of MDL-12330A rapidly caused all well activity to cease with increasing doses of PE (*n* = 1–10). Interestingly, a nonsignificant decrease in activity was observed in response to MDL-12330A in the presence of 1 μM PE (Fig. 5*B*), whereas 1 μM PE alone did not result in a change. These experiments have been previously performed using isolated guinea pig atrial myocytes (25, 59); however, this is the first time that these experiments have been performed using human-derived cells, and these results highlight the potential translational relevance of this signaling pathway in humans.

## DISCUSSION

The IP_3_ signaling pathway plays an important role in the control of cardiac activity, in regulating both inotropy and cardiac pacemaker activity (20, 23, 25, 39). Furthermore, increasing evidence highlights the involvement of IP_3_ signaling in cardiac arrhythmogenesis. However, the mechanisms involved are not yet fully understood (21, 25, 36, 46).

In both atrial and ventricular cells, L-type Ca^2+^ current (*I*_Ca,L_) has been shown to undergo regulation by cytosolic Ca^2+^ (74). In ventricular cells, this regulation may be primarily modulated via CaMKII, as shown by the inhibition of *I*_Ca,L_ by KN-93 (74); however, in atrial myocytes, the addition of the Ca^2+^ selective chelator BAPTA, following KN-93, results in further inhibition of *I*_Ca,L_ not observed in ventricular myocytes, suggesting the presence of a CaMKII-independent mechanism of Ca^2+^ regulation (74). These effects of BAPTA are reversed following the addition of exogenous cAMP to the cell (74). One potential mechanism that has been proposed to explain these observations, is the activation of Ca^2+^-sensitive ACs, downstream of IP_3_R Ca^2+^ release (25, 74). The Ca^2+^-sensitive AC isoforms AC1 and AC8 both show expression in cardiomyocytes, including atrial cardiomyocytes and the SAN cells (25, 57).

An alternative mechanism that has been proposed for linking IP_3_R Ca^2+^ release with effects on CaT amplitude and contractility is that Ca^2+^ released from IP_3_R leads to an increase in [Ca^2+^] within the immediate vicinity of RyRs, which, in turn, enhances the response of RyRs to Ca^2+^ influx via LTCCs, resulting in an amplification of CICR (23, 56). In isolated atrial cells, however, the enhancement of Ca^2+^ signal resulting from either pharmacological G_q/11_-coupled receptor stimulation, or direct cytosolic release of caged IP_3_, is inhibited following antagonism of ACs by MDL-12330A, or PKA by H89 (25), and our data in the present study also support the presence of this pathway in human cells, as shown by the inhibition of the response to PE in hiPSC-ACMs (Fig. 5). It is not possible to directly confirm that these effects are related to activation of cAMP in response to PE from these experiments alone, but the reduced response in the presence of H89 does provide support for the involvement of PKA in the beating rate response to PE in human cells. H89 only partially affected the response to higher concentrations of PE (Fig. 5*B*). This result was expected, however, and consistent with our conclusions because we used a dose of H89 that only causes partial PKA inhibition (75), to avoid off-target effects, and cAMP regulates cardiac pacemaking independent of PKA through HCN (76–78) and POP-EYEDC (79). Although LTCCs are regulated by AC5 and AC6 (1, 80), and *I*_Ca,L_ is inhibited by MDL-12330A (74), the direct sensitization of RyRs to Ca^2+^ released from IP_3_R would be expected to persist in the presence of AC inhibition. However, MDL-12330A and H89 both result in a complete inhibition of the effects of IP_3_R Ca^2+^ release on the CaT (25), indicating the involvement of AC activation downstream of IP_3_R activation and before the involvement of RyRs. Interestingly, we observed a nonsignificant drop in beating rate in response to MDL-12330A in the presence of 1 μM PE despite not observing an increase in activity in experiments, where 1 μM PE was used alone or in the presence of H89 (Fig. 5*B*). MDL-12330A is a nonspecific AC inhibitor, therefore, likely to reduce basal [cAMP], therefore disrupting the coupling of the Ca^2+^ and membrane clock (81–83) and thus the baseline pacemaking rate as well as changes in pacemaking responses (5, 6, 84). Previous studies have shown that the effect of adenylyl cyclase inhibition on pacemaking can be reversed by membrane-permeant cAMP (84), suggesting the effects are through changes in cAMP. It is possible that increased SR Ca^2+^ release following IP_3_R activation could impair the pacemaking in the absence of this IP_3_R-mediated Ca^2+^ release stimulating a rise in cAMP since the Ca^2+^ release might not occur in vicinity with NCX channels. This increased SR Ca^2+^ leak may impair synchronized local Ca^2+^ release events and CaTs through reducing SR Ca^2+^ content, causing Ca^2+^-dependent inactivation of adjacent RyR2s or LTCCs, leading to a shallower delayed depolarization slope and thus pacemaking. We cannot rule out the possibility that the effect of MDL-12330A on the response to PE in hiPSC-ACMs is because of the effect of MDL-12330A on the basal [cAMP] and the subsequent inhibition of pacemaking mechanisms and other pathways. However, partial inhibition of AC1 by ST-034307 significantly reduces the positive chronotropic effects of PE in spontaneous beating murine right atria without affecting the baseline beating rate (25, 59). These data support the idea that the inhibition by MDL-12330A of the increase in the frequency of spontaneous action potentials by PE in hiPSC-CMs is feasible due to a role of ACs that is specific to the pacemaking response to PE versus an independent effect on pacemaking. The effect of inhibition and modulation of the activity of different ACs in human adult or hiPSC-derived atrial cardiomyocytes on pacemaking response to PE, however, remains to be investigated in the future.

In the present study, we have shown using the EPAC-S^H187^ FRET sensor that cytosolic cAMP rises in response to pharmacological activation of α-AR and direct application of membrane-permeant IP_3_ (Fig. 2). The rise in cAMP was inhibited following the addition of the IP_3_R inhibitor 2-APB (Fig. 2*C*). However, it has been reported that the effects of 2-APB on inhibiting Ca^2+^ release are related to a direct inhibitory effect on TRPC channels rather than inhibition of IP_3_R (67). In addition, we cannot rule out the potential of direct stimulation of TRPC following IP_3_R Ca^2+^ release (23). For this reason, we repeated experiments using the alternative IP_3_R inhibitor Xestospongin-C and again observed an inhibition of rise in cAMP in the presence of this IP_3_R antagonist (Fig. 2*E*). Potential mechanisms that could account for these changes in cAMP in response to IP_3_R Ca^2+^ release are discussed next.

**Figure 6.**
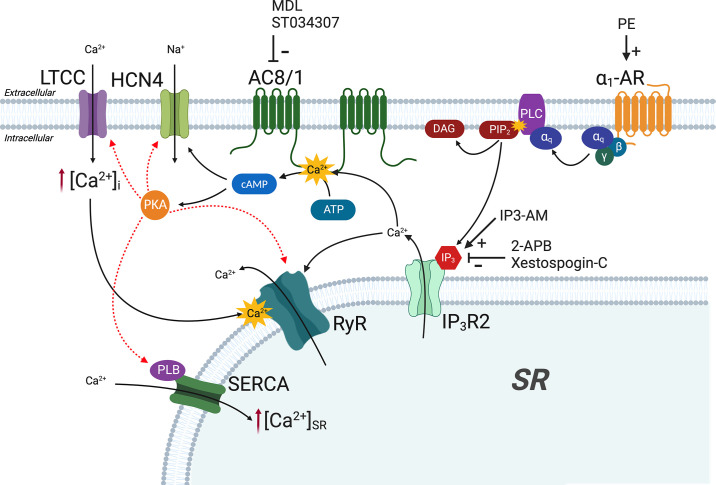
Scheme indicating potential mechanism presented in discussion for changes in cyclic adenosine monophosphate (cAMP) in response to inositol trisphosphate (IP_3_) receptor 2 (IP_3_R_2_) Ca^2+^ release. The scheme indicates potential mechanisms by which activation of α_1_-adrenergic receptors (α_1_-ARs) by phenylephrine (PE) may lead to increased atrial cytoplasmic [Ca^2+^] transient (CaTs; indicated by intracellular [Ca^2+^]) and cAMP production based on published observations in addition to our current results. Activation of α_1_-AR leads to elevated IP_3_ resulting from cleavage of phosphatidylinositol-4,5 biphosphate (PIP_2_) to diacylglycerol (DAG) and IP_3_ by phospholipase C (PLC). IP_3_ activation of IP_3_R_2_ results in release of Ca^2+^ from the sarcoplasmic reticulum (SR), which subsequently leads to activation of Ca^2+^-sensitive adenylyl cyclases (AC8 or AC1) and activation of protein kinase A (PKA) by cAMP, or direct effects of cAMP on the funny current (*I*_f_). In this proposed scheme, AC8/AC1 are placed in the sarcolemma. MDL, MDL-12330A. Images created with a licensed version of BioRender.com.

1) Ca^2+^ released via IP_3_R may directly lead to the activation of Ca^2+^-sensitive AC isoforms AC1 and/or AC8 in cardiac cells. Although such a link has not yet been directly shown in atrial cells, in the SAN, selective inhibition of Ca^2+^-sensitive AC1 using ST034307 results in a decrease in spontaneous beating rate (59), whereas chelation of Ca^2+^ using BAPTA and inhibition of ACs using MDL-12330A reduce the hyperpolarization-activated “funny” current (*I*_f_) (74). These data support the involvement of AC1 stimulation following IP_3_R Ca^2+^ release in the regulation of SAN pacemaker activity (25, 39, 57, 59). As the coexpression of AC1 and RyR2 in the SAN (59) appears to be comparable with that of AC8 and RyR2 in atrial cells (25), it is possible that a similar link between IP_3_R Ca^2+^ release and activation of AC8 is present in atrial cardiomyocytes. Overexpression of AC8 in mouse cardiac cells has been shown to result in increased contractility, independent of *I*_Ca,L_, demonstrating that endogenous AC8 may have the ability to influence cardiac contractility via a mechanism independent of AC5/6 (85). Furthermore, NRAMs expressing AKAP79-CUTie presented an increase in FRET signal on the addition of PE (Fig. 4), highlighting the possibility that IP_3_R Ca^2+^ release could be activating AC8 and/or AC1 because of its proximity. Further details on this proposed mechanism are shown in Fig. 6.
2) IP_3_R Ca^2+^ release has been shown to enhance uptake of Ca^2+^ into lysosomes (86), which themselves may act as an important intracellular Ca^2+^ store, influencing cardiac Ca^2+^ handling (87, 88). This process is itself regulated via cAMP (86, 89, 90). By boosting lysosomal [Ca^2+^], this process would be expected to enhance lysosomal Ca^2+^ release by the action of NAADP on lysosomal two-pore channel 2 (TPC2) (88).
3) Ca^2+^ release via IP_3_R may lead to activation of store-operated Ca^2+^ channels (SOCCs), such as TRPC or Orai channels, most likely being TRPC3, as they are located on the surface membrane of SAN cells (23, 91). In addition, there is strong evidence for a functional interaction between TRPC3 (and TRPC6/7), IP_3_R, and capacitive Ca^2+^ entry (91–93). Activation of SOCCs in response to IP_3_ could be expected to result in the downstream activation of Ca^2+^-sensitive AC1/8. In HEK293 cells, heterologous overexpression of AC1 and AC8 results in functional colocalization of AC1/8 with SOCCs (94), whereas, endogenously, AC8 and AC5/6 can be regulated by store-operated Ca^2+^ entry (SOCE) (95, 96).
4) cAMP may be generated by activation of AC downstream of enhanced RyR Ca^2+^ signals following IP_3_R Ca^2+^ release. As discussed earlier, this pathway appears unlikely in the models tested here due to the inhibition of changes in the CaT in response to IP_3_ signaling in the presence of MDL-12330A and H89 (25); however, additional experiments involving the inhibition of RyRs would be required to confirm this.

Our data comparing the rise in cytosolic cAMP in response to PE in atrial cells versus ventricular cells highlight the absence of inhibition of the cAMP signal in the presence of either 2-APB or Xestospongin-C (Fig. 3*B*) in ventricular myocytes, indicating that this rise likely occurs independently of IP_3_R Ca^2+^ release in ventricular myocytes, in contrast to our findings in atrial myocytes. These different results in atrial versus ventricular myocytes are likely explained by a combination of differences between atrial and ventricular myocytes. IP_3_Rs are expressed at significantly higher levels in atrial myocytes than in ventricular myocytes (20), including NRAMs versus NRVMs, suggesting that the quantity of Ca^2+^ released by IP_3_Rs is likely less in ventricular myocytes and thus unable to stimulate AC1 or AC8. Moreover, IP_3_Rs in NRVMs are localized perinuclear regions (97, 98), thus meaning that the Ca^2+^ released by these IP_3_Rs will not be in the correct location to stimulate AC1 or AC8, which are likely located in the sarcolemma based on studies in adult cardiomyocytes (25, 57, 59, 74). Overall, NRAMs have more IP_3_Rs than NRVMs, and these IP_3_Rs are likely located in puncta toward the periphery of cells based on adult rodent atrial myocytes (20, 25, 59), meaning that they can likely release sufficient Ca^2+^ from IP_3_Rs in the correct locations to stimulate AC1 or AC8, which are also more prevalent. Markers involved in Ca^2+^ handling such as LTCCs and RyRs are differentially expressed between atrial and ventricular myocytes (3), meaning that Ca^2+^ handling and diffusion is different between atrial and ventricular myocytes. These data support the presence of an IP_3_R-dependent pathway of cAMP activation that is present in atrial myocytes but absent or reduced in ventricular myocytes.

Several FRET-based sensors rely on the binding of AKAPs, principally AKAP79, for targeting subcellular domains (17). AKAP79 binds to the dimerization/docking domain of PKA RII subunits (99) and is localized to the plasma membrane through binding with PIP_2_ (100). In addition, AKAP79 can interact with PKC (101) and Ca^2+^-dependent protein phosphatase 2B (17, 102). In cardiomyocytes, AKAP79 forms a complex with β-AR, AC5/6, and LTCC (17, 103). One potential drawback of the use of AKAP79-targeted FRET sensors is the possibility that these sensors may themselves impact the kinetics and amplitude of the local changes in cAMP. However, the AKAP79-CUTie FRET sensor has been shown to avoid these issues (17). Using AKAP79-CUTie, we observed changes in FRET within the region of the plasma membrane in response to IP_3_R activation via potentiation of G_q/11_-coupled receptors, using PE. AKAP79-CUTie has been previously used to identify cAMP nano-domain signaling within the region of β-AR (16), and given the association of AKAP79 with AC5/6, we cannot rule out the possibility that the cAMP changes which we observed resulted from the action of these AC isoforms. AC5/6 are inhibited by Ca^2+^ (85). However, our experiments using 2-APB and Xestospongin-C (Fig. 4) demonstrate that the changes we observed in cAMP using AKAP79-CUTie are dependent on either IP_3_R Ca^2+^ release or SOCCs. Therefore, AC8 would be a more likely candidate for this rise in cAMP, as we know that *I*_Ca,L_ in atrial cardiomyocytes may also be regulated by Ca^2+^-dependent ACs (74) and that AC8 is expressed in this region of the plasma membrane (25, 59).

Although *I*_Ca,L_ and RyR2 are strongly suspected as the downstream targets of IP_3_-cAMP signals, the direct phosphorylation and activation by PKA in response to IP_3_ of these or any other target proteins has yet to be shown. Moreover, a direct role for IP_3_-cAMP signals in the reported proarrhythmic effects of IP_3_ signaling in atria has yet to be proven, although it appears likely if RyR2 and *I*_Ca,L_ are phosphorylated by PKA following IP_3_ signaling. Although the amplification of the CaT in atrial myocytes via IP_3_ is PKA dependent (25), this does not mean IP_3_ cAMP signals cannot have alternative proarrhythmic effects on atrial myocytes via other cAMP-sensitive targets such as HCN, EPAC1/2, POP-EYE domain-containing proteins, and phosphodiesterase 10. In addition, the effects of cAMP-hydrolyzing phosphodiesterases that may regulate the IP_3_-cAMP remain unknown.

PE is predominantly considered to act through α_1_-adrenergic agonism, including in atrial cardiac myocytes (25), stimulating IP_3_ production through a G_q/11_-PLC-β pathway. Landzberg et al. (104) showed that in humans, PE exerts a positive inotropic effect that is inhibited by the α-AR antagonist phentolamine, demonstrating the selectivity of PE in human cardiac tissue. We believe that the stimulation of α_1_-adrenergic receptors is the most likely explanation for our results. However, we cannot rule out the possibility that PE is stimulating β-adrenergic signaling directly (105) or indirectly (106) and causing a subsequent rise in cAMP. To minimize the potential indirect stimulation of β-ARs by PE-induced release of norepinephrine from neurons (105), we cultured cells from neonatal rat atria in a cardiomyocyte maintenance media. The rapid rise in cAMP over a short time frame also suggests a direct action of PE on our imaged cells against an indirect mechanism. We have found that the rise in cAMP following the addition of PE appears to be IP_3_R dependent, suggesting that PE is most likely acting through an α_1_-receptor-G_q/11_-PLC-IP_3_ signaling pathway. Although, it is not impossible that PE may be stimulating IP_3_ signaling through β-adrenergic signaling (107, 108). In previous publications, to minimize any β-adrenergic component, we used the β-AR inhibitor metoprolol at 1 µM in conjunction with PE (25). Therefore, although we cannot rule out the involvement of β-AR activation in response to PE without genetic knockout experiments, the rise in cAMP observed in response to IP_3_-AM (Fig. 1, *E* and *F*), alongside the inhibition of the response to PE by IP_3_R inhibitors, does demonstrate that this cAMP rise occurs following direct activation of IP_3_R.

In this article, we have shown for the first time, using FRET-based sensors (EPAC-S^H187^ and AKAP79-CUTie), that cytosolic cAMP levels are increased in atrial myocytes following α_1_-AR agonist (Fig. 1). In atrial cells, these changes in cAMP can be inhibited by either 2-APB or Xestospongin-C, confirming that cAMP is generated subsequently to IP_3_R activation in atrial cardiomyocytes. Whether this rise in cAMP occurs as the result of the direct influence of Ca^2+^ release from IP_3_R on Ca^2+^-sensitive AC1/8 or via an intermediate effect, such as activation of SOCE, remains to be determined. Conclusive identification of the targets of IP_3_-cAMP signals, any proarrhythmic effects, and the cAMP phosphodiesterases that regulate IP_3_-cAMP signals in atrial myocytes may identify novel targets for the modulation of proarrhythmic atrial IP_3_ signaling.

## DATA AVAILABILITY

Data will be made available upon reasonable request.

## SUPPLEMENTAL MATERIAL

10.6084/m9.figshare.26335852.v1 Supplemental Figs. S1–S4: https://doi.org/10.6084/m9.figshare.26335852.v1.

## GRANTS

This study was supported by British Heart Foundation (BHF) Project Grants PG/18/4/33521 (to S.J.B.) and RG/17/6/32944 (to M.Z.) and Wellcome Trust (WT) Grant 109371/Z/15/Z (to R.-A.A.B.B. and R.A.C.). R.-A.A.B.B. received research funds from the Ellis T. Davies Fellowship Endowment, University of Liverpool, and from Covid-19 Rebuilding Research Momentum Fund (CRRMF) Oxford Funds and Returning Carers Funding (Oxford). T.A. received funding from the Returners Carers Fund (principal investigator, R.-A.A.B.B.), Medical Science Division, University of Oxford, the Nuffield Benefaction for Medicine, and the WT Institutional Strategic Support Fund (ISSF), University of Oxford. E.A. received funding from the Returners Carers Fund (principal investigator, R.A.C.), University of Oxford.

## DISCLOSURES

S.D.B. and R.A. were employees of Axol Bioscience, Ltd. (Easter Bush, UK), the manufacturers of the iPSC-derived atrial cardiomyocytes and related reagents used in this study. None of the other authors has any conflicts of interest, financial or otherwise, to disclose.

## AUTHOR CONTRIBUTIONS

D.A.T. and R.-A.A.B.B. conceived and designed research; E.C.A., M.J.R., S.J.B., R.A.C., Y.-C.C., T.A., S.D.B., R.A., and J.N.S. performed experiments; M.J.R., S.J.B., A.K., R.A.C., Y.-C.C., M.F., T.A., S.D.B., R.A., J.N.S., D.A.T., M.Z., and R.-A.A.B.B. analyzed data; S.J.B., Y.-C.C., M.F., T.A., S.D.B., R.A., J.N.S., D.A.T., M.Z., and R.-A.A.B.B. interpreted results of experiments; E.C.A., S.J.B., T.A., S.D.B., and R.-A.A.B.B. prepared figures; E.C.A., S.J.B., R.A., and R.-A.A.B.B. drafted manuscript; E.C.A., M.J.R., S.J.B., A.K., R.A.C., Y.-C.C., M.F., T.A., S.D.B., R.A., J.N.S., D.A.T., M.Z., and R.-A.A.B.B. edited and revised manuscript; E.C.A., M.J.R., S.J.B., A.K., R.A.C., T.A., S.D.B., J.N.S., D.A.T., M.Z., R.-A.A.B.B. approved final version of manuscript.
